# Open‐source deep‐learning models for segmentation of normal structures for prostatic and gynecological high‐dose‐rate brachytherapy: Comparison of architectures

**DOI:** 10.1002/acm2.70089

**Published:** 2025-04-05

**Authors:** Andrew J. Krupien, Yasin Abdulkadir, Dishane C. Luximon, John Charters, Huiming Dong, Jonathan Pham, Dylan O'Connell, Jack Neylon, James M. Lamb

**Affiliations:** ^1^ Department of Radiation Oncology University of California Los Angeles California USA

**Keywords:** Auto‐contouring, brachytherapy, dice‐similarity‐coefficient, segmentation

## Abstract

**Background:**

The use of deep learning‐based auto‐contouring algorithms in various treatment planning services is increasingly common. There is a notable deficit of commercially or publicly available models trained on large or diverse datasets containing high‐dose‐rate (HDR) brachytherapy treatment scans, leading to poor performance on images that include HDR implants.

**Purpose:**

To implement and evaluate automatic organs‐at‐risk (OARs) segmentation models for use in prostatic‐and‐gynecological computed tomography (CT)‐guided high‐dose‐rate brachytherapy treatment planning.

**Methods and materials:**

1316 computed tomography (CT) scans and corresponding segmentation files from 1105 prostatic‐or‐gynecological HDR patients treated at our institution from 2017 to 2024 were used for model training. Data sources comprised six CT scanners including a mobile CT unit with previously reported susceptibility to image streaking artifacts. Two UNet‐derived model architectures, UNet++ and nnU‐Net, were investigated for bladder and rectum model training. The models were tested on 100 CT scans and clinically‐used segmentation files from 62 prostatic‐or‐gynecological HDR brachytherapy patients, disjoint from the training set, collected in 2024. Performance was evaluated using the Dice‐Similarity‐Coefficient (DSC) between model predicted contours and clinically‐used contours on slices in common with the Clinical‐Target‐Volume (CTV). Additionally, a blinded evaluation of ten random test cases was conducted by three experienced planners.

**Results:**

Median (interquartile range) 3D DSC on CTV‐containing slices were 0.95 (0.04) and 0.87 (0.09) for the UNet++ bladder and rectum models, respectively, and 0.96 (0.03) and 0.88 (0.10) for the nnU‐Net. The rank‐sum test did not reveal statistically significant differences in these DSC (*p* = 0.15 and 0.27, respectively). The blinded evaluation scored trained models higher than clinically‐used contours.

**Conclusion:**

Both UNet‐derived architectures perform similarly on the bladder and rectum and are adequately accurate to reduce contouring time in a review‐and‐edit context during HDR brachytherapy planning. The UNet++ models were chosen for implementation at our institution due to lower computing hardware requirements and are in routine clinical use.

## 1 INTRODUCTION

Minimization of dose to healthy tissue is important throughout radiation oncology to reduce toxicity effects. Treatment planning requires the delineation of organs‐at‐risk (OAR) for dose‐volume optimization, and in traditional radiation therapy workflows, the OAR delineation process is often done manually.^1^, ^2^ This is costly time‐and‐labor‐wise for organizations, and automated segmentation has been a goal of radiation oncology research for over two decades. In recent years, deep learning has revolutionized auto‐segmentation research, and radiation oncology clinics are now deploying commercially^3^ or locally^4^ trained deep‐learning models to auto‐contour various OARs^5^ and speed up treatment planning workflows.^6^

For prostatic and gynecological HDR brachytherapy treatments, treatment planning acceleration is especially pragmatic as the dose is delivered through temporarily implanted applicators. Planning images are differentiated from external beam radiotherapy (EBRT) images by the presence of catheters and applicators, which can shift tissue, create image artifacts, and reduce image quality.^7^, ^8^ Despite recent research into creating and implementing autosegmentation models for brachytherapy planning procedures, to the best of our knowledge, there are few models commercially available and none publicly available, and research efforts lack training on large and diverse datasets for use in both prostatic and gynecologic CT‐based HDR brachytherapy planning procedures.^6^, ^9^, ^10^, ^11^, ^12^ Choosing a quality auto‐segmentation model for training is important, especially when working with images that contain objects that shift tissue and artifacts that disrupt the quality of the images. Recent papers exploring and evaluating the implementation of autosegmentation models for HDR brachytherapy treatment planning tend to employ the UNet^13^ model or a variation of it through a popular pre‐configured autosegmentation model training method, the nnU‐Net.^14^ The nnU‐Net (no‐new‐U‐Net) method adapts to the properties of a given training dataset and enables the training of four different UNets: 2D, 3D fullres, 3D lowres, and 3D cascade fullres. We chose to include UNet++^15^ in this study due to prior success with the model in related tasks,^4^ and we chose to include the 2D nnU‐Net model due to usage and performance in other HDR brachytherapy autosegmentation studies.^6^, ^10^, ^11^ Additionally, 2D networks are associated with faster prediction speeds and greater GPU compatibility and model generalizability.^6^, ^16^

Our training and test batches include both prostatic and gynecological cases and, therefore, provide our models with greater performance capabilities and applicability for a wider range of cases, increasing their practicality and potential for clinical use. To the best of our knowledge, this is the first complete work to evaluate deep learning‐based autosegmentation models trained on both prostatic and gynecological HDR brachytherapy treatment planning data. Furthermore, to the best of our knowledge, this is the first work in the realm of autosegmentation for brachytherapy to include lower quality Mobile CT Scanner data in model training, and we simultaneously use the largest and most diverse dataset to date. Worthy of note, images from the Airo‐Mobile CT Scanner (Stryker, Inc., Portage, MI) tend to be lower quality due to increased presence of streaks and artifacts.^17^, ^18^

This paper presents a comprehensive evaluation of novel deep learning‐based autosegmentation models for prostatic and gynecological CT‐based HDR brachytherapy. We first describe our datasets and methodology, then provide training details, and finally, our results describe test performances. The UNet++ model was chosen for implementation at our institution due to lower computing hardware requirements and is in routine clinical use. Trained models and implementation code have been made publicly available.

## 2 MATERIALS & METHODS

### 2.1 Clinical data selection

A total of 1416 pelvic HDR brachytherapy simulation CTs and structure files (RTStructs) from prostatic and gynecological treatments performed from 2017 to 2024 were retrospectively collected for training and testing. Images were acquired using the following scanners: Airo‐Mobile CT (Stryker, Portage, MI), SOMATOM Drive, Sensation Open, Sensation 64, SOMATOM Definition AS (Siemens Medical Systems, Erlangen, Germany), or Brilliance Big Bore (Philips, Cambridge, MA). Details of the distribution of data between scanner and treatments are listed in Table 1. The slice thickness of the scans was always 2 mm, the image matrix size was 512 × 512 pixels, and the pixel spacings ranged from 0.21875 to 1 mm. The clinically‐used planning images and bladder and rectum contours, contoured for treatment planning by experienced dosimetrists, medical physicists, and radiation oncologists, were extracted from the CTs and RTStructs and used for model training and testing. The contouring process was performed on MIM contouring software, versions 6.14–7.3.5 (MIM Software Inc. Cleveland, OH).

**TABLE 1:** Description of dataset.

		Training	Hold‐out test
Patients		1105	62
CT scans		1316	100
Slices		169 937	9.902
	Sensation open	727	0
	Airo‐mobile	551	91
	Sensation 64	14	0
	Brilliance big bore	17	0
	SOMADefinition AS	2	0
	SOMATOM drive	5	9
Treatment type	Prostatic	765	53
	Gynecological	551	47

*Note*: Distribution of data between scanner and treatment types. The table includes the number of prostatic and gynecologic patients, scans, and slices in the training and hold‐out test sets.

### 2.2 Data processing

CTs and RTStructs were exported as DICOM files from MIM and axial slices were transformed into numpy images and structure masks of matrix size 512 × 512 pixels for training. Images were normalized to contain values between one and zero. The nnU‐Net extracts a dataset fingerprint (a set of dataset‐specific properties such as image sizes, voxel spacings, intensity information, etc.) and uses this information to design three U‐Net configurations, each operating on its own preprocessed version of the dataset and designed to work out‐of‐the‐box. For ease of training with UNet++ without a preprocessing pipeline automatically configured to the dataset, contrast enhancement through scikit‐image's Contrast Limited Adaptive Histogram Equalization (CLAHE) method and downsampling to 128 × 128 pixel^2^ was performed to increase the availability of information to the models, reduce computational cost, and decrease model training time.^19^, ^20^ These preprocessing steps were not performed for the nnU‐Net model because the nnU‐Net model includes its own preprocessing steps.^14^ The same preprocessing steps used for training were applied to CTs for testing.

### 2.3 Training details

**FIGURE 1:**
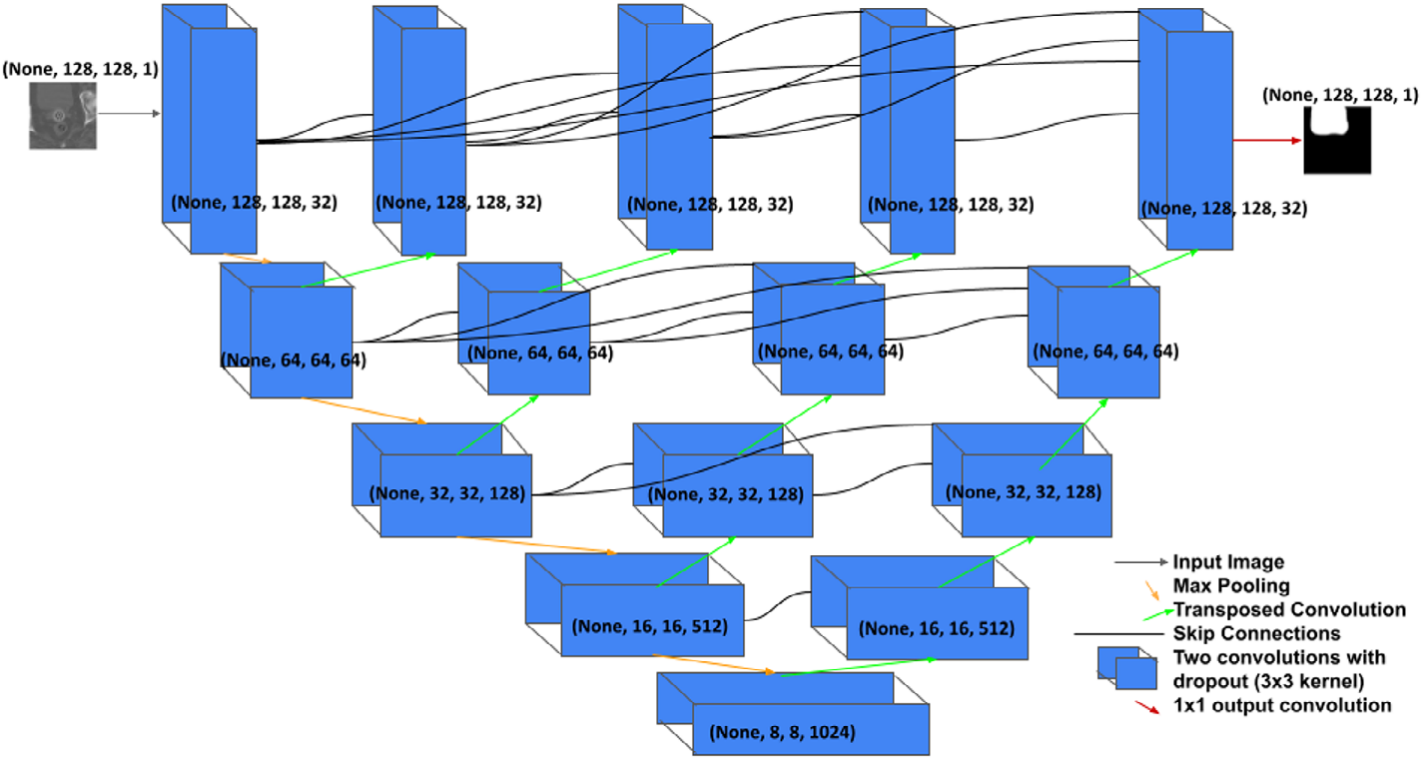
UNet++ diagram. Black curves indicate Skip Connections, orange arrows Max Pooling operations, green arrows Transposed Convolutions, and the blue boxes two convolutions followed by a dropout layer. The dropout layers are represented with the two convolutions as the blue boxes.

For the UNet++ models, after random shuffling, a 90‐10 split was used to divide the training dataset (Table 1) into training and validation sets. Data augmentation was performed every epoch by applying random rotations between –10° and 10° and image zooms between 0% and 20%. The Adam optimizer from Keras was utilized with a learning rate of 8 × 10^−5^ and a batch size of 32. This learning rate was chosen empirically as greater values overshot the minimum and led to instability in training, and smaller learning rates could not fit the task and failed to train. The filters ran from [32,64,128,512,1024]. The kernel size was 3 × 3, and the stride: 2 × 2. The kernel initializer: “he normal”, the convolutional layer activation: “relu”, the output layer activation: “sigmoid”, after two convolutional layers the dropout = 0.3, and the convolutional layer padding: “same.” Below in Figure 1 is a diagram of the UNet++ architecture. We define the loss function for the UNet++ using the Dice Similarity Coefficient as it has previously proved reliable and effective for training auto‐segmentators.^4^, ^21^

For the 2D nnU‐Net models, after normalization, each sample's numpy images and masks were stacked and transformed into Nifti arrays for training. Random sampling is applied by nnU‐Net to create an 80–20 split to divide the training dataset into training and validation sets. The nnU‐Net uses a combination of the DSC and cross‐entropy as a loss function and does its own data‐augmentations, including random rotations, random scaling, random elastic deformation, gamma correction, and mirroring. The 3D nnU‐Net models were too large for our GPU available for clinical computing purposes, as the system failed to allocate enough memory on the GPU to perform the task, which has 12GB of RAM. Keras with Tensorflow (versions 2.15.0) and Python (3.11.7) were used for training UNet++, and PyTorch (2.3.0) was used for training the 2D nnU‐Net models. All trainings were completed using either one or four NVIDIA GeForce GTX Titan X 980 GPUs with 12GB of Memory each. The 2D nnU‐Net models, and the UNet++ bladder model were trained using one GPU. The UNet++ rectum model was trained using all four GPUs. In this case, the batch size was increased from 32 to 128 to compensate for the increased computation capacity.

### 2.4 Performance evaluation

Performance accuracy was characterized by DSC in three ways: first, 3D DSC provided a global similarity measure. Second, 3D DSC computed on clinically‐used contours and auto‐contours were restricted by masking to only slices containing clinical target volume (CTV) contours to mitigate the confounding effects of variability in superior‐inferior extensions of clinical contours outside the region where they would impact treatment plan dosimetry. Third, 2D DSC were computed on every slice that contained CTV contours in an attempt to quantify the fraction of slices that could be used for planning with minimal editing. Finally, 10 random samples were chosen from the test set to be visually evaluated by three experienced treatment planners on a scale from one through ten. Planners were asked to evaluate bladder and rectum contours from both UNet++ and nnU‐Net models and also clinically‐used contours. The origin of the contours (nnU‐Net, UNet++, or clinically‐used) was blinded from evaluators during the rating process. On this scale, a rating of ten required no modifications, a rating of seven required minor modifications, a rating of four required major modifications, and a rating of one was unusable.

## 3 RESULTS

**FIGURE 2:**
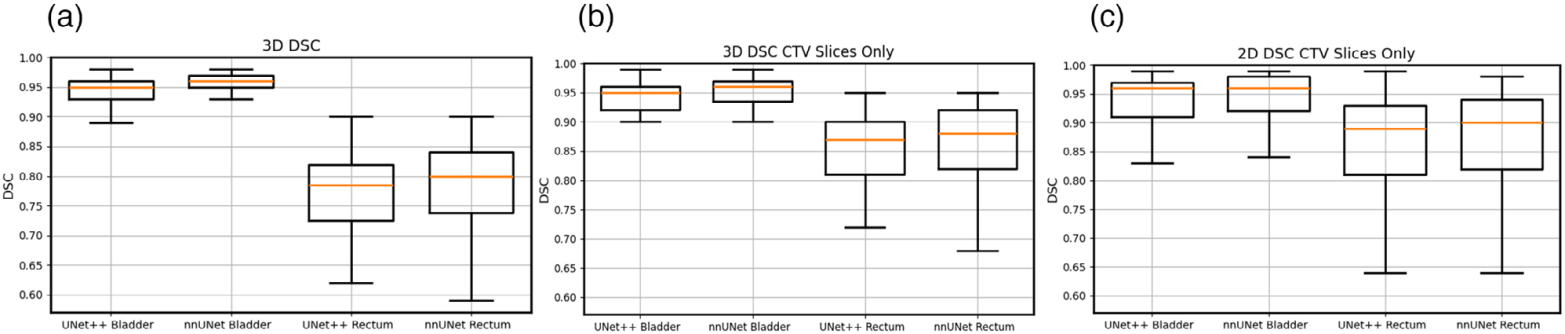
DSC evaluation of the autocontours. Autocontours are compared to clinically‐used contours. Plot A represents the DSC evaluated across the 3‐Dimensional volume, Plot B represents the DSC evaluated across the 3‐Dimensional volume restricted to CTV slices only, and Plot C represents the DSC evaluated on a 2D slice by slice basis, restricted to CTV slices only. Box‐and‐whisker plots show median, interquartile range, and the whiskers extend from the box to the farthest data point lying within 1.5x the inter‐quartile range (IQR) from the box.

**FIGURE 3:**
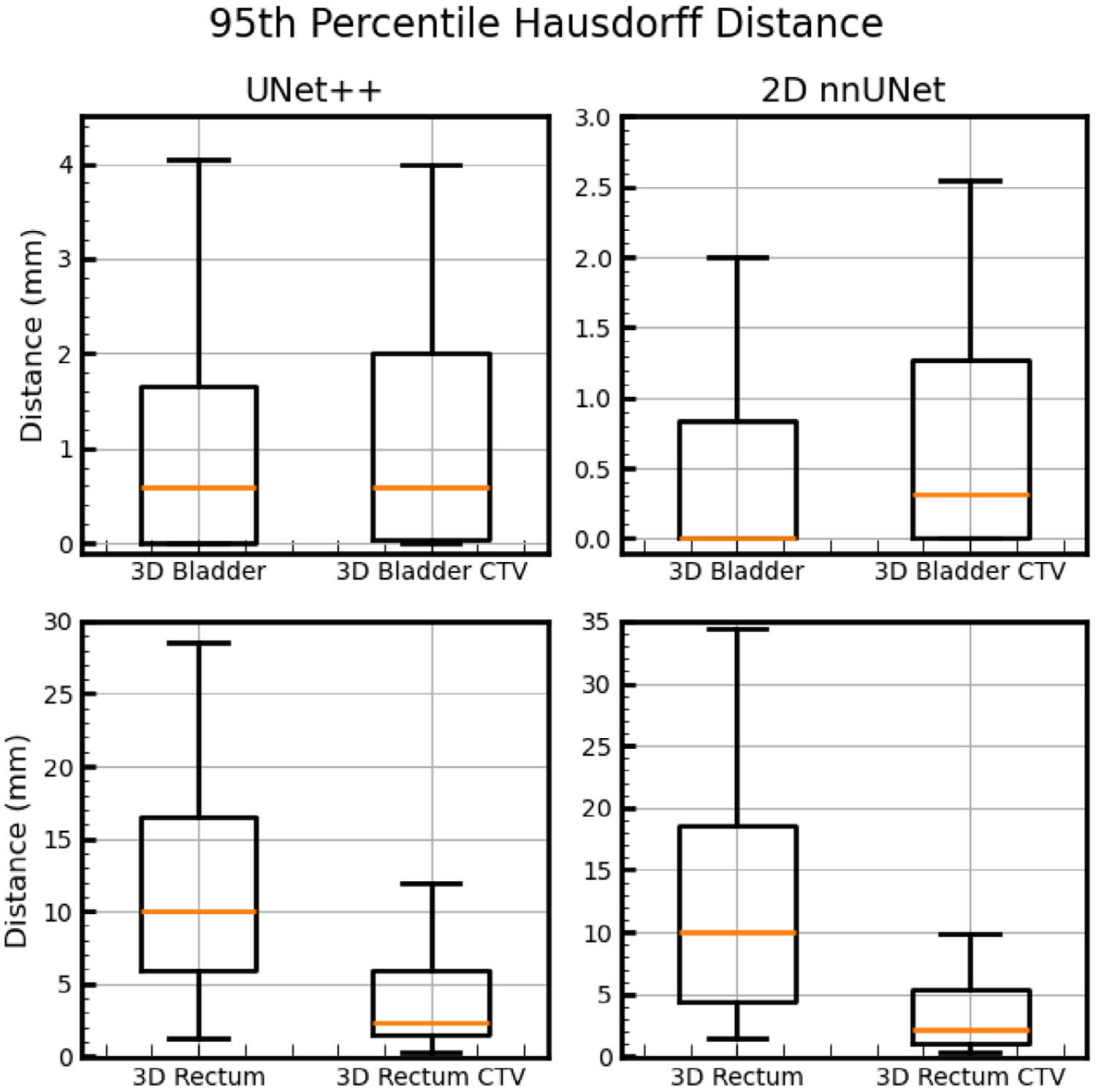
HD 95^th^ Percentile evaluation of the autocontours. Autocontours are compared to clinically‐used contours. Box‐and‐whisker plots show median, interquartile range, and the whiskers extend from the box to the farthest data point lying within 1.5x the inter‐quartile range (IQR) from the box.

**FIGURE 4:**
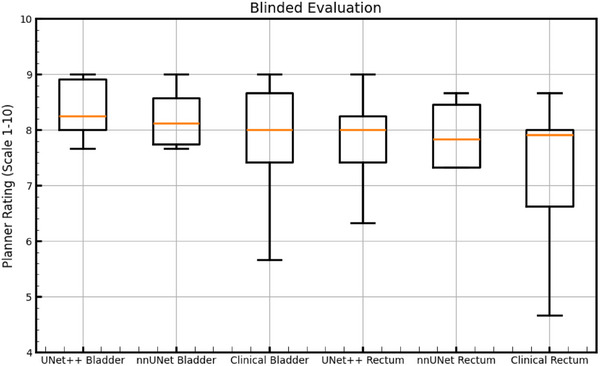
Results from the blinded evaluation of the auto‐ and clinically‐used contours. Box‐and‐whisker plots show median, interquartile range, and the whiskers extend from the box to the farthest data point lying within 1.5x the inter‐quartile range (IQR) from the box. On this scale, a rating of ten required no modifications, a rating of seven required minor modifications, a rating of four required major modifications, and a rating of one was unusable.

**FIGURE 5:**
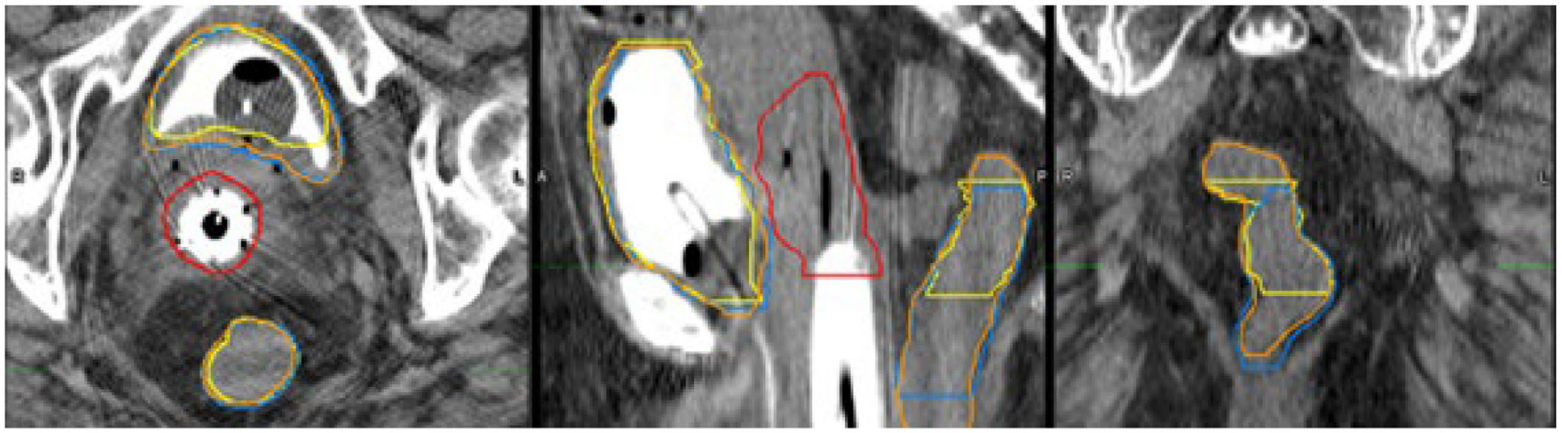
Tri‐Plane view of minimum 3D rectum dice for UNet++. 0.32 for UNet++ and 0.43 for 2D nnU‐Net. Clinically‐used contours are in Yellow. UNet++ autocontours are in Blue, and 2D nnU‐Net autocontours are in Orange. The clinically‐used CTV is in Red. This figure shows the lowest global 3D rectum DSC for the UNet++ model. This figure further illustrates the variability in model predictions with respect to the vertical position of the rectum.

**FIGURE 6:**
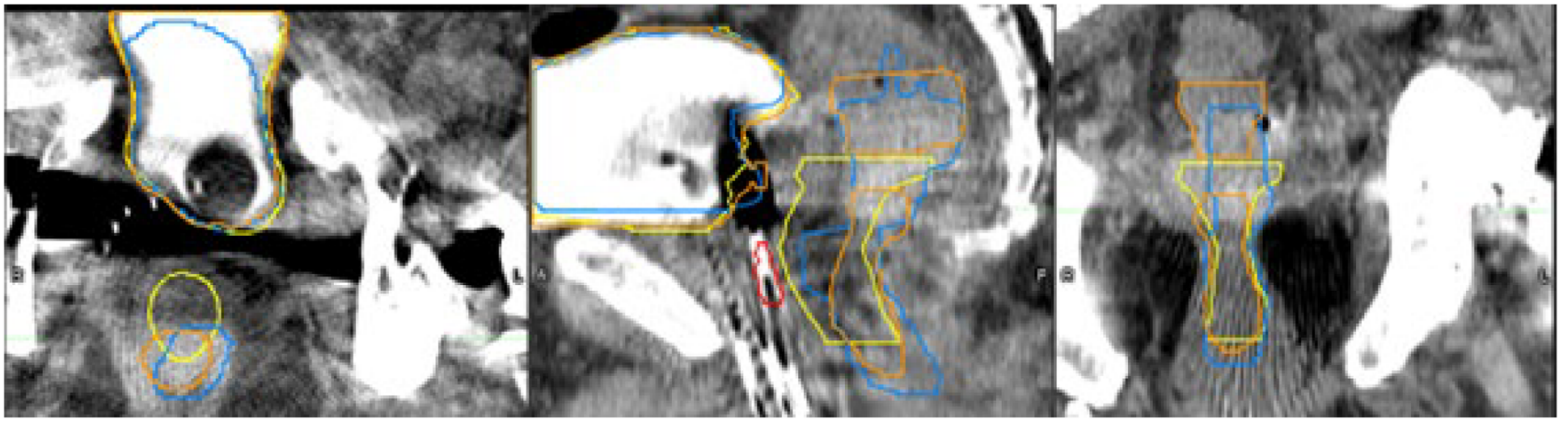
Tri‐Plane view of minimum 3D rectum dice for 2D nnU‐Net. 0.23 for 2D nnU‐Net and 0.36 for UNet++. Clinically‐used contours are in Yellow. UNet++ autocontours are in Blue, and 2D nnU‐Net autocontours are in Orange. The clinically‐used CTV is in Red. Model performance suffered in the presence of major photon starvation artifacts. The height of the rectum was ambiguous, and for the 2D nnU‐Net model the rectum was disconnected.

**FIGURE 7:**
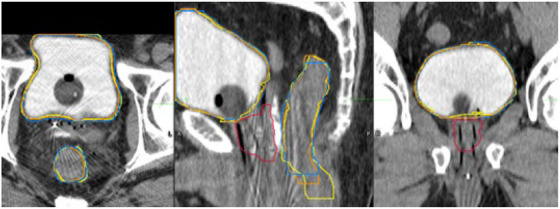
Tri‐Plane view of maximum 3D bladder dice of 0.98 for both networks. Clinically‐used contours are in Yellow. UNet++ autocontours are in Blue, and 2D nnU‐Net autocontours are in Orange. The clinically‐used CTV is in Red. The models performed well despite the presence of minor streaking artifacts frequently present in Airo scans.

**FIGURE 8:**
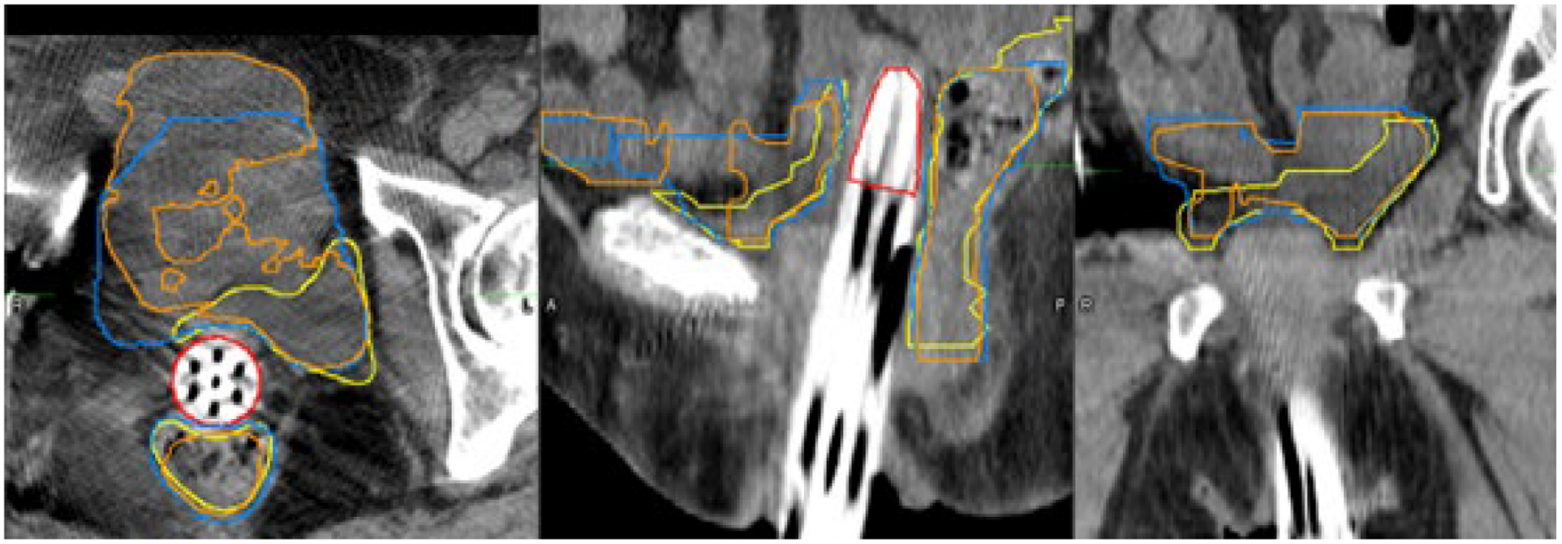
Tri‐Plane view of minimum 3D bladder dice of 0.45 for UNet++ and 0.41 for 2D nnU‐Net. Clinically‐used contours are in Yellow. UNet++autocontours are in Blue, and 2D nnU‐Net autocontours are in Orange. The clinically‐used CTV is in Red. The models did not perform as well in the presence of large insertions leading to anatomy deformation.

Figure 2 below shows DSC results for 100 test cases comparing clinically‐used contours to auto‐contours from the UNet++ and 2D nnU‐Net models. Figure 3 following depicts 3D HD95th Percentile results. From Figure 2 readers can see rectum contours DSC benefitted more than bladder contours DSC from evaluation on CTV slices and evaluation in 2D on CTV slices. Figure 3 shows the rectum benefitted more than the bladder from evaluation on CTV slices in terms of the HD95th percentile. According to our experienced planner's visual evaluations represented in Figure 4, our UNet++ models outperformed both the nnU‐Net models, and the dosimetrist final contours. The UNet++ bladder and rectum models scored a median of 8.25 and 8, respectively, and the nnU‐Net bladder and rectum models scored a median of 8.1 and 7.8, respectively. While the dosimetrist final bladder and rectum contours scored a median of 8 and 7.9, respectively. A table describing all test results by machine and a figure of the MDA results are included in Supplementary Information. Figures 5, 6, 7, 8 following provide select autocontour examples from both models overlaid with clinically‐used contours.

When looking at Figures 5 and 6, the rectum autocontours include more axial slices than the clinically‐used contours. The clinically‐used contours do not include the entirety of the rectum and there may be some ambiguity as to the axial positions of the rectum. The 3D DSC may return “unfair” results in these cases and not accurately represent model performance, so a 2D DSC or DSC focusing on CTV slices only may more accurately represent model performance. For both networks, the rectum DSC values show greater improvement than the bladder DSC when only considering slices in common with the CTV. This was expected, as in our clinical practice, the entire bladder was almost always segmented in its entirety regardless of proximity to the CTV, whereas the rectum was always segmented where proximal to the CTV, but the superior‐inferior extent of rectum segmentation beyond the level of the CTV was highly variable. This is well‐illustrated by Figure 5 which shows the lowest global 3D rectum DSC for the UNet++ model. Of note, not all test cases had bladder clinically‐used contours with axial slices in common to the CTV. Amongst the 100 scans in the test set, there were four cases where there was no bladder on axial slices in common with the CTV.

## 4 DISCUSSION

Delineation of OARs throughout radiation oncology is crucial to reduce dose to healthy tissue and spare patients from toxicity‐effects. Manually contouring OARs for cervical cancers has been measured in cervical brachytherapy treatment planning to take an average of 14 minutes, whereas editing auto‐contours for use has been shown to only take an average of 2.1 minutes, and preprocessing, prediction, and network transfer using the nnU‐Net model only took an average of 1.6 minutes.^6^ Run‐time is hardware and image dependent in addition to being algorithm dependent. The time of prediction for our UNet++ models with their configuration and our NVIDIA GeForce GTX Titan X 980 GPU is on average 0.84 seconds per 3D image without including preprocessing and network transfer latency. The objective of this study was to train diverse auto‐contouring models on large datasets and measure their accuracy in pelvic brachytherapy cases that are diverse in terms of disease treated and CT scanner used for planning. We further sought to evaluate the performance of the networks in a “review and edit” framework by additionally analyzing the dice coefficient on axial slices common with the Clinical Target Volume, and in 2D on axial slices, as this should provide a greater gauge of model performance near clinical target volumes and also when there is less ambiguity on if rectum or bladder is present in an axial slice.

To the best of our knowledge, we use the largest and most diverse dataset to date for training autosegmentation models for CT‐based HDR brachytherapy planning. Given our models consider greater data variability, it is difficult to properly compare our test results to those from studies using less diverse datasets. Our median DSC values were on par with those in related studies which achieved mean or median DSC values in the range of 0.94–0.87 for the bladder and 0.84–0.82 for the rectum.^6^, ^9^, ^10^, ^11^, ^12^ Our results, in consideration of slices containing CTV contours, meet but do not exceed these performance levels. Nevertheless, we believe our results represent an advance over previous work because we included both prostatic and gynecological cases and included a majority of testing data from the Airo Mobile CT scanner, which has a demonstrated tendency toward lower image quality due to streaking artifacts.^17^, ^18^ Our models performed well in the presence of the minor streaking artifacts frequently present in Airo scans (e.g., Figure 7) but suffered in the presence of major photon starvation artifacts (as in Figure 6) and irregular anatomy (as in Figure 8).

The two model architectures trained show great similarity in performance between each other on the test set, but the 2D nnU‐Net models may slightly outperform the UNet++ models quantitatively. For example, all DSC results were non‐normal according to the Shapiro–Wilk tests, and the T Test revealed statistically significant differences between models only in the 2D Bladder CTV slice DSC results (*p* = 0.04) while the rank‐sum test revealed statistically significant differences for all DSC results except for the 3D Rectum DSC and the 3D Rectum CTV slice DSC (*p* = 0.15, and *p* = 0.27, respectively). There were no statistically significant differences in variances according to Levene's test.

We implemented the UNet++ models into our treatment planning services. Our models used an in‐house developed DICOM server to recognize incoming CT scans by label, auto‐contour these scans, and send them to MIM so that they are available to treatment planners for use to produce clinically used contours. When in use, the models are explicitly timesaving as treatment planners do not have to create contours from scratch. We have observed person‐to‐person differences in the willingness of dosimetrists to use autocontours, consistent with previous investigations of barriers and facilitators to clinical automation.^22^ Further investigation of factors affecting clinical impact are the subject of future work.

Some limitations to our study pertain to our training processes: with our available GPUs, we were unable to successfully train 3D nnU‐Net models, which might have obtained better performance. Furthermore, in training our models, we were limited in our capabilities to explore all possible combinations of parameters due to time and computational power constraints and therefore did not necessarily fully explore whether we reached maximum trainable model capacity given the large size of our training dataset. Another limitation in the evaluation of our models is that the test set is primarily composed of Airo CT scans, whereas the training dataset is more balanced in origin. However, this reflects the current practice at our institution to prioritize the use of the Airo scanner and, furthermore likely presents the greatest challenge to the algorithm because of lower scan quality. Finally, the greatest limitation of our study may be the use of clinical contours without curation. Data lacking curation can lead to lower‐quality datasets that may contain points of error and limit our model training performance, or our ability to assess model performance. However, with greater than 1000 scans used, manual curation is infeasible. Data auto‐curation is a current topic of research but outside the scope of the present paper. Future work should investigate the use of foundation contouring models such as Segment Anything Model (SAM), SAM 2, TotalSegmentator, and MedSAM.^16^, ^23^, ^24^, ^25^, ^26^ These models have been trained on large quantities of data which allows them to exist as general models for a variety of tasks. Future works may seek to see if better performance can be achieved through transfer learning or fine‐tuning such foundation models on smaller, fully curated clinical datasets for particular and specific tasks. Future works may also seek to see if performance can be maintained or improved through transfer learning into larger, and more general models applicable to more tasks.

## 5 CONCLUSION

In summary, we successfully provide deep learning‐based models trained and tested on 1416 clinically‐used CTs and RTStructs for autosegmentation of the bladder and rectum in prostatic and gynecologic HDR brachytherapy planning. The 2D nnU‐Net models statistically slightly outperformed the UNet++ configuration, but performance was deemed equivalent for clinical purposes. Deep learning tools like this one can save time and effort. We successfully provide a new tool for autosegmentation in HDR brachytherapy and have done so with training data encompassing prostatic and gynecological treatment data to increase the tool's usability. The UNet++ model was chosen for implementation at our institution due to lower computing hardware requirements and is in routine clinical use. Trained models and implementation code have been made publicly available.

## AUTHOR CONTRIBUTIONS

The study was conceptualized by James Lamb, Yasin Abdulkadir, and Andrew Krupien. Dishane Luximon, Dylan O'Connell, and Jack Neylon contributed to hardware organization and support. Andrew Krupien and Yasin Abdulkadir contributed to data collection and model training. Andrew Krupien retrieved results and calculated statistical values. Jonathan Pham, Huiming Dong, and John Charters contributed to visual evaluations. Andrew Krupien was responsible for writing the paper. All authors participated in the discussion of results and revisions to the manuscript.

## CONFLICT OF INTEREST STATEMENT

The authors declare no conflicts of interest.

## Supporting information



Supporting Information

## Data Availability

The data that support the findings of this study are available from the corresponding author upon reasonable request and are subject to institutional review board review and UCLA administrative requirements.
