# Supplementary material for: Open‐source deep‐learning models for segmentation of normal structures for prostatic and gynecological high‐dose‐rate brachytherapy: Comparison of architectures
Source: J Appl Clin Med Phys. 2025 Apr 5;26(6):e70089. doi: 10.1002/acm2.70089 (PMC12148797; doi:10.1002/acm2.70089)
Supplement: Supplementary file 1 — Supporting Information [file ACM2-26-e70089-s001.doc]

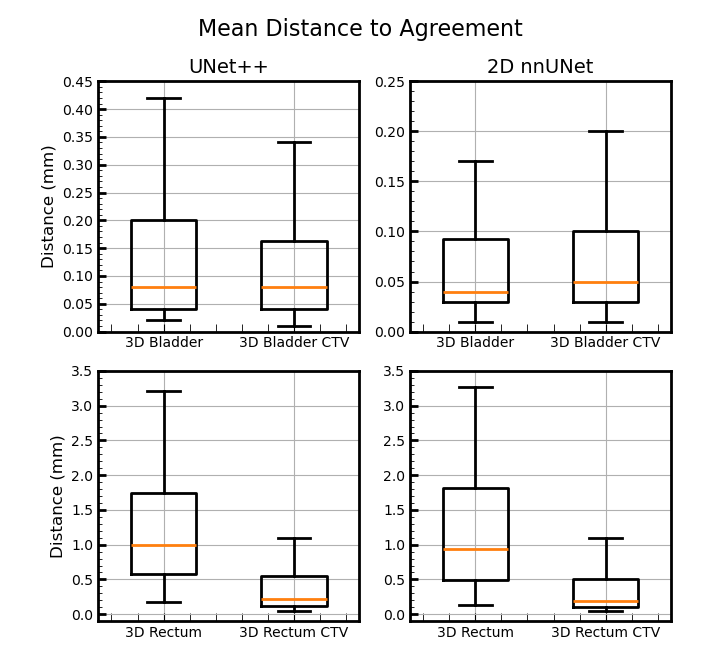


**Supplementary Figure**

MDA evaluation of the autocontours. Autocontours are compared to clinically-used contours. Box-and-whisker plots show median, interquartile range, and the whiskers extend from the box to the farthest data point lying within 1.5x the inter-quartile range (IQR) from the box.

| Organ | Metrics | Airo-Mobile  Unet++ | Airo-Mobile  2d nnUNet | SOMATOM Drive  Unet++ | SOMATOM Drive  2d nnUNet |
| --- | --- | --- | --- | --- | --- |
| Bladder | 3D DSC | .95 ± .08 | .97 ± .08 | .91 ± .05 | .94 ± .03 |
|  | 3D DSC CTV Slices Only | .95 ± .21 | .96 ± .23 | .91 ± .15 | .92 ± .12 |
|  | 2D DSC CTV Slices Only | .96 ± .25 | .97 ± .12 | .93 ± .32 | .95 ± .14 |
|  | 95^th^ Percentile HD (mm) | .44 ± 5.36 | 0.0 ± 5.57 | 2.05 ± 7.36 | 1.97 ± 2.44 |
|  | 95^th^ Percentile HD CTV Slices Only (mm) | .59 ± 3.94 | .31 ± 4.22 | 2.21 ± 4.63 | 2.0 ± 2.92 |
|  | MDA (mm) | .08 ± .81 | .04 ± .92 | .22 ± .72 | .15 ± .15 |
|  | MDA CTV Slices Only (mm) | .06 ± .68 | .05 ± 1.33 | .23 ± .45 | .16 ± .40 |
| Rectum | 3D DSC | .78 ± .1 | .80 ± .10 | .79 ± .14 | .79 ± .10 |
|  | 3D DSC CTV Slices Only | .87 ± .12 | .88 ± .10 | .86 ± .09 | .90 ± .10 |
|  | 2D DSC CTV Slices Only | .89 ± .22 | .91 ± .11 | .90 ± .25 | .91 ± .10 |
|  | 95^th^ Percentile HD (mm) | 10.0 ± 9.42 | 10.0 ± 9.15 | 12.26 ± 14.86 | 15.09 ± 10.66 |
|  | 95^th^ Percentile HD CTV Slices Only (mm) | 2.35 ± 4.56 | 2.13 ± 5.66 | 4.0 ± 5.82 | 2.11 ± 4.12 |
|  | MDA (mm) | 1.0 ± 1.81 | .88 ± .1.34 | .98 ± 2.77 | 1.21 ± 1.37 |
|  | MDA CTV Slices Only (mm) | .22 ± .63 | .19 ± .63 | .48 ± .48 | .2 ± .37 |

**Supplementary Table**

Median test results and standard deviations reported by CT Scanner. MDA and HD results are reported in millimeters. Dice scores are reported on a scale from 0 to 1, with 1 being a perfect match.

**Supplementary Link:** [**https://gitlab.com/akrupien/hdrpac**](https://gitlab.com/akrupien/hdrpac)

A link to the open-source implementation code for autocontouring DICOM images sent over a DICOM network and the trained models.
